# Acute jejunoileal obstruction due to a pseudopolyp in a child with undiagnosed crohn disease: A case report

**DOI:** 10.1186/1752-1947-2-54

**Published:** 2008-02-20

**Authors:** Efstratios Christianakis, Nikolaos Pashalidis, Stavroula Kokkinou, Michael Pitiakoudis, Evangelos Mplevrakis, Maria Chorti, Spiros Rizos, Dimitrios Filippou

**Affiliations:** 1 Department of Pediatric Surgery, Pendeli's Children Hospital, Athens, Greece; 2 First Department of Surgery, Piraeus General Hospital "Tzaneio", Piraeus-Athens, Greece; 3 Cytogenetic Unit, Sismanoglio General Hospital, Athens, Greece; 4 Department of Pathology Sismanoglio General Hospital, Athens, Greece; 5 Department of Anatomy, University of Athens, Nursing Faculty, Athens, Greece

## Abstract

**Introduction:**

Crohn's disease (CD) can affect any part of the alimentary tract from the mouth to the anus, with most common site being the terminal ileum.

**Case presentation:**

A child suffering from undiagnosed Crohn disease (CD), presented with an acute abdominal obstruction due to a large pseudopolyp in the jejunoileal area. At laparotomy, a jejunoileal segment of 45 cm, containing multiple areas of damage to the small intestine, was excised and a primary end – to – end anastomosis was performed.

**Conclusion:**

The coexistence of an intestinal pseudopolyp with undiagnosed Crohn's disease may be the cause of acute abdominal obstruction in children.

## Introduction

Crohn's disease (CD) can affect any part of the alimentary tract from the mouth to the anus, with most common site being the terminal ileum. Bowel obstruction is a well-known complication of CD, usually as the result of stricture formation, or more rarely as mechanical obstruction. Intestinal obstruction due to a large pseudopolyp is a rare event in CD [1,2].

## Case presentation

**Figure 1:**
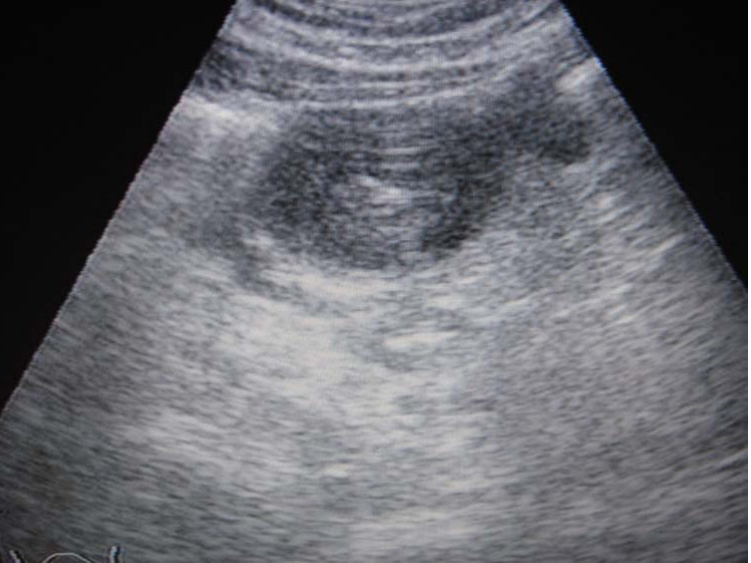
Preoperative ultrasound showing the large pseudopolyp in the jejunoileal region.

A 12-year-old boy was brought to the emergency department with acute abdominal pain lasting 12 hours, and associated abdominal distension, absolute constipation for two days, vomiting and fever of 38.5°C. When examined he had general abdominal tenderness. White blood cell count was 17.5 k/ml with 85.5% neutrophils, hemoglobin was 10.9 gr/dl, hematocrit 34.7% and platelets 820 k/ml. Abdominal X-rays showed air-fluid levels. Abdominal ultrasound examination revealed a solid intraluminal pattern (Figure 1). The patient gave a history of referred intermittent abdominal pain for a period of 6 months. More detailed clinical information, such as diarrhea for the past 6 months, quick tiredness, no mood to play, lethargy and paleness, was obtained postoperatively.

**Figure 2:**
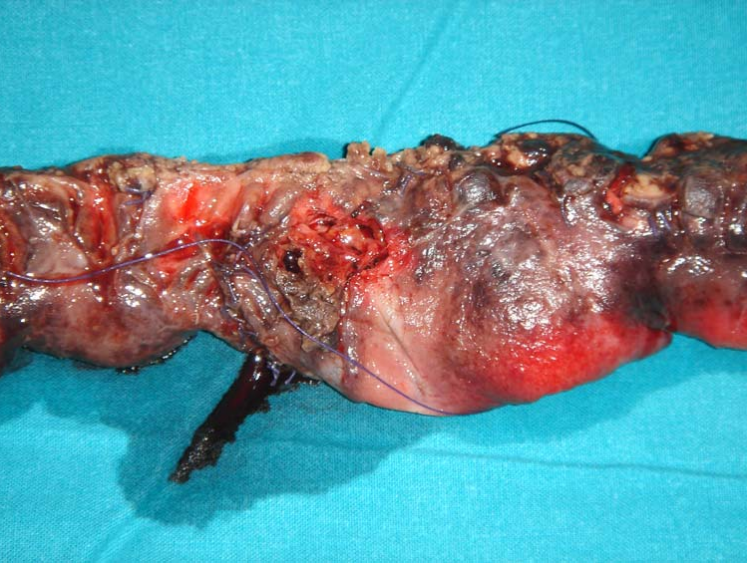
Photograph of the surgical specimen.

The patient underwent laparotomy and 30 cm of small bowel with multiple areas of damage was found. The damage included macroscopically a rigid and thickened mass in the ileal portion, creeping fat, multiple granulomas in the external intestinal surface and ulcers, two of which had parietal ruptures with fluid escape. A resection of 45 cm of the ileo-jejunal portion, including all areas of intestinal damage, was performed and a primary end to end ileo-jejunal anastomosis completed the operation (Figure 2). Longitudinal incision of the intestine showed a cobblestone appearance, due to linear ulcers crossing with transverse folds. Linear ulcers were created from interconnected rows of aphthous ulcers. A characteristic large pseudopolyp, 4 cm in diameter, was in the obstructed portion of the mass.

**Figure 3:**
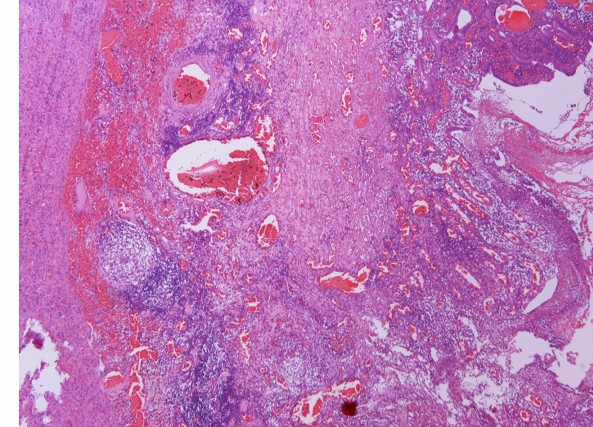
Histological examination of the specimen revealed oedema and diffuse inflammation throughout the whole intestinal wall (C,D).

Microscopically, edema and diffuse inflammation of the whole intestinal wall, fissures, granulomas, vascular dilatation, pseudopolyps, mucosal inflammation of the small and large bowel, and granulomas in local lymph nodes were observed. The tip of the appendix was inflamed too (Figure 3).

One month later, endoscopic examinations showed granulomas and other Crohn's lesions in the stomach and colorectum.

The patient was treated with 1.5 g daily dose of Mesalamine for one year, without recurrence of the disease. During this period he also received Modulen complementary oral feeds and his growth was normal.

## Discussion

CD can affect any part of the alimentary tract from the mouth to the anus, with the most common site being the terminal ileum [1,2]. Approximately 15% of all patients with CD are children. There are special pathological features that distinguish CD from Ulcerative Colitis [3]. A cobblestone appearance is not uncommon, due to linear ulcers crossed with transverse folds. Linear ulcers are created from interconnected rows of aphthous ulcers. CD is not cured surgically [1]. More than 50 % of children with CD require surgery because of complications, failure of medical therapy or growth failure. Growth failure is a common manifestation that is the result from both the decreased caloric intake of the inflammatory bowel and the circulation of inflammatory cytokines [2]. Complications of CD include intestinal obstruction because of strictures, intestinal perforation, bleeding or fistulas. The main goal of surgical therapy is the removal of damaged bowel, maintaining the maximal amount of intestine possible. Other possible operations include strictureplasty without bowel resection, segmental or subtotal colectomy, and proctocolectomy with Brooke ileostomy. Proximal diversion alone does not secure healing of the excluded segments of bowel [3].

It is not unusual to find segmental CD and frequently the rectum is spared of disease. Bowel obstruction is a well-known complication of CD usually as the result of stricture formation or more rarely as mechanical obstruction. Intestinal obstruction due to a large pseudopolyp is a rare event in CD. These types of pseudopolyps rarely regress with medical management alone, often requiring surgical resection [4]. There have been two different types of pseudopolyps described in adult CD, one form that in seen in the large intestine in Crohn colitis and a second form which is the nodular lymphangiectasia occurring in the small intestine [5]. There have not been any descriptions of small intestine pseudopolyps in children before.

Lastly, genetic testing of our patient showed a deletion of p53 and ATM genes and the presence of the rearrangement of BCL6 gene. This means that he is at high risk of developing a cancerous disease and may also develop malignant lymphoma and many other types of cancer and solid tumours [6].

## Conclusion

The coexistence of an intestinal pseudopolyp with undiagnosed Crohn's disease may be the cause of acute abdominal obstruction in children.

## Competing interests

The author(s) declare that they have no competing interests.

## Authors' contributions

EC, EM, DF operated on the patient, MC and SK performed the diagnostic and histological examinations, MP, NP and SR participated in the follow up and the diagnostic strategy. All authors participated in writing the case report and revising the draft.

## Consent

Written informed consent was obtained from the patient and his parents for publication of this Case report and accompanying images. A copy of the written consent is available for review by the Editor-in-Chief of this journal.
